# Preventive Strength of Dyadic Social Interaction against Reacquisition/Reexpression of Cocaine Conditioned Place Preference

**DOI:** 10.3389/fnbeh.2017.00225

**Published:** 2017-11-08

**Authors:** Tanja Bregolin, Barbara S. Pinheiro, Rana El Rawas, Gerald Zernig

**Affiliations:** Experimental Psychiatry Unit, Department of Psychiatry 1, Medical University of Innsbruck (MUI), Innsbruck, Austria

**Keywords:** dyadic social interaction, cocaine, dependence syndrome, addiction, substance abuse disorder, C57BL/6J mouse, C57BL/6N mouse, Sprague-Dawley rat

## Abstract

The reorientation away from drugs of abuse and toward social interaction is a highly desirable but as yet elusive goal in the therapy of substance dependence. We could previously show that cocaine preferring Sprague-Dawley rats which engaged in only four 15 min episodes of dyadic social interaction (DSI) did not reacquire and reexpress cocaine conditioned place preference (CPP) after a single cocaine exposure. In the present study, we investigated how strong this preventive effect of DSI is. In corroboration of our previous findings in rats, four 15 min DSI episodes prevented the reacquisition/reexpression of cocaine CPP in mice. However, this effect was only observed if only one cocaine conditioning session (15 min) was used. If mice were counterconditioned with a total of four cocaine sessions, the cocaine CPP reemerged. Interestingly, the opposite also held true: in mice that had acquired/expressed cocaine CPP, one conditioning session with DSI did not prevent the persistence of cocaine CPP, whereas four DSI conditioning sessions reversed CPP for 15 mg/kg intraperitoneal cocaine. Of note, this cocaine dose was a strong reward in C57BL/6J mice, causing CPP in all tested animals. Our findings suggest that both the reversal (reconditioning) of CPP from cocaine to DSI as well as that from DSI to cocaine requires four conditioning sessions. As previously shown in C57BL/6 mice from the NIH substrain, mice from the Jackson substrain also showed a greater relative preference for 15 mg/kg intraperitoneal cocaine over DSI, whereas Sprague-Dawley rats were equally attracted to contextual stimuli associated with this cocaine dose and DSI. Also in corroboration of previous findings, both C57BL/6J mice and experimenters several generations removed from the original ones produced CPP for DSI to a lesser degree than Sprague-Dawley rats. Our findings demonstrate the robustness of our experimental model across several subject- and experimenter generations in two rodent genus (i.e., mouse and rat) and allow the quantification of the strength (i.e., persistence) of the preventive effect of DSI against the reacquisition/reexpression of cocaine CPP, arguably a model for cocaine relapse.

## Introduction

The reorientation away from drugs of abuse and toward social interaction is a highly desirable but as yet mostly elusive goal in the therapy of substance dependence (Zernig et al., 2007, 2013; Zernig and Pinheiro, 2015), warranting its neurobiologic investigation. The main reason for performing the present experiments was our seminal finding (Fritz et al., 2011b) that counterconditioning with dyadic (i.e., one-to-one) dyadic social interaction (DSI) of previously cocaine-preferring Sprague-Dawley rats not only reversed their conditioned place preference (CPP) for cocaine, but that these rats also defended their CPP for DSI against cocaine in spite of a final single re-exposure (conditioning session) with cocaine (arguably a model for cocaine relapse after experiencing a “freebie”). Our rat findings were corroborated by other independent groups using our paradigm (Yates et al., 2013) or other experimental approaches involving DSI (Peartree et al., 2012).

Now, many would argue that such a remarkable preventive/protective effect of social interaction against cocaine relapse could hardly be found in human addicts who are often impaired in their social interaction and find interacting with others more aversive than non-drug-dependent individuals (see e.g., Zernig et al., 2000, 2003; Nutt et al., 2010). At the animal experimental level, the mouse genus is considered by many researchers to be less “social” or “prosocial” than rats, i.e., exhibits a simpler social behavioral repertoire and much less social flexibility (see e.g., Whishaw et al., 2001). Accordingly, we had demonstrated that C57BL/6N mice spent less time in direct physical contact with each other and found DSI—associated contextual stimuli less attractive than rats (Kummer et al., 2014; Pinheiro et al., 2016). If one accepts that “much less social flexibility” in the mouse may model “impaired social interaction” in human addicts to some degree, then the mouse genus would confer greater translational power to animal experimentals on DSI as a non-drug alternative to drugs of abuse.

The mouse is also a more attractive animal experimental model than the rat because many more addiction research-relevant transgenic models are available in this genus and the wealth of data already generated with transgenic models in the mouse is considerably larger than that in rats. To illustrate, a pubmed search on “(d1 or drd1)” and “transgenic” and “mouse” yielded 1028 hits, whereas the same search profile for “rat” gave only 100 hits (accessed on 25 August, 2017; with dopamine D1 receptor expressing medium spiny neurons being prime candidates for the modulation of motivated behavior including addictive behavior).

In particular, we explored if the preventive effect of DSI against reacquisition/reexpression of cocaine CPP would also persist under repeated exposure to cocaine in the mouse model. We investigated this because in the course of our parametric studies, it turned out that a higher percentage of C57BL/6N mice than Sprague-Dawley rats found DSI aversive rather than attractive (Kummer et al., 2014). We also found that the conditioned aversion to DSI-associated stimuli was more pronounced in the NIH substrain (C57BL/6N) than the Jackson substrain (C57BL/6J; Zernig and Pinheiro, 2015; Pinheiro et al., 2016) of C57BL/6 mice. We expected the protective effect of DSI conditioning in C57BL/6J mice to dissipate within four sessions of counterconditioning with cocaine. For the reasons given above, we did not address this question in the rat model.

The present findings show that DSI conditioning should be long enough (i.e., four 15 min episodes in a cocaine-free environment) to countercondition previously cocaine-conditioned C57BL/6J mice and to protect them against the subsequent reacquisition/reexpression of cocaine CPP. The present results also demonstrate that even in DSI counterconditioned mice, a subsequent cocaine history that is long enough (i.e., four 15 min cocaine conditioning sessions) is sufficient to overcome the protective effect of DSI. Taken together, our results indicate that currently abstinent but previously cocaine dependent individuals have to be exposed to drug-free social interaction continuously for abstinence to be maintained. Suggestions derived from the present findings for the design of experiments investigating the neural basis of DSI- vs. cocaine preference are given below.

## Materials and Methods

### Subjects

Male C57BL/6J mice (8 weeks old, weighing 22–23 g) and early adult male Sprague-Dawley rats (200–250 g, corresponding to an age of 8 weeks) were obtained from Charles River Laboratories (Sulzfeld, Germany). No information was available which animals were littermates or cagemates. All animals were delivered in group transport cages and singly housed after receipt. This study was carried out in accordance with the recommendations of the Austrian Federal Ministry of Science, Research and Economy (Bundesministerium fuer Wissenschaft, Forschung und Wirtschaft, BMWFW). The protocol was approved by the Animal Experiment Ethics Committee of the BMWFW.

### Place Conditioning Procedure

#### Housing Conditions and CPP Apparatus

All animals were singly housed at a constant room temperature of 22°C and had *ad libitum* access to tap water and pelleted chow (Tagger, Austria). Experiments were performed during the light phase of a continuous 12 h light/dark cycle with the lights on from 08:00 h to 20:00 h. Before the start of the CPP experiments, animals were singly housed for 5–7 days and experienced a total of seven 2 min handling episodes with their allocated experimenter (at least one handling episode per day).

Conditioning was conducted in a three compartment apparatus (CPP box 64 cm wide × 32 cm deep × 31 cm high) made of unplasticized polyvinylchloride. The middle (neutral) compartment (10 × 30 × 30 cm) had white walls and a white floor. Two doorways led to the two conditioning compartments (25 × 30 × 30 cm each) with walls showing either vertical or horizontal black-and-white stripes of the same overall brightness and with stainless steel floors containing either 168 holes (diameter 0.5 cm) or 56 slits (4.2 × 0.2 cm each). Time spent in each compartment was digitally recorded with a video camera and analyzed offline with hand timers. The CPP apparatus was cleaned with a 70% camphorated ethanol solution after each session. All experiments were performed under neon ceiling light (58 W, 1 m distance) and white noise from continuously running allergen filter boxes.

#### Experimental Groups

**Figure 1:**
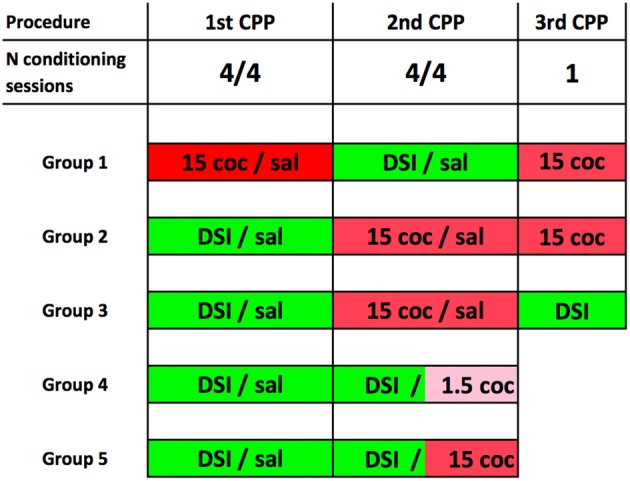
Experimental timeline for place preference conditioning to dyadic social interaction (DSI) vs. cocaine. The timeline of the behavioral training is shown here in schematic form. 15 coc, 15 mg/kg pure base intraperitoneal (i.p.) cocaine, injected as HCl salt in saline; 1.5 i.p. coc, 1.5 mg/kg cocaine. Sal, saline injected in a volume of 10 ml/kg in mice and 1 ml/kg in rats. DSI, always preceded by an i.p. saline injection. Groups 1–3 consisted of C57BL/6J mice only. Please note that in group 4 (C57BL/6J mice only) and group 5 (C57BL/6 mice or Sprague-Dawley rats), the 2nd conditioned place preference (CPP) procedure was a concurrent one, i.e., that the DSI stimulus was pitched directly against the cocaine stimulus (Zernig and Pinheiro, 2015). Group sizes were always *N* = 8 animals. See “Materials and Methods” section for details.

The schematic experimental timeline of the experiment is shown in Figure 1. Our conditioning procedure has been described and discussed in detail previously (Fritz et al., 2011b; Zernig et al., 2013; Prast et al., 2014; Zernig and Pinheiro, 2015). For the acquisition of CPP for DSI or cocaine (coc) with saline (sal) injections serving as vehicle/handling control, the conditioning procedure comprised a pretest session on day 1, followed by eight consecutive training days in an alternate-day-design of the pattern DSI-sal-DSI-sal-DSI-sal-DSI-sal or coc-sal-coc-sal-coc-sal-coc-sal, respectively (one training session per day, e.g., cocaine on Monday, saline on Tuesday, cocaine again on Wednesday and so on). CPP/CPA was tested on day 10, i.e., 48 h after the last cocaine dose (and 24 h after the last saline injection). For conditioning to DSI (vs saline), the stimuli were either: (1) a 15 min DSI session with a sex- and weight-matched male conspecific preceded by an intraperitoneal (i.p.) saline injection in a volume of 10 ml/kg saline in mice or 1 ml/kg in rats; or (2) only a saline injection as the comparator stimulus. For cocaine (coc) conditioning (vs sal), the test mouse was either: (1) injected i.p. with 1.5 or 15 mg/kg pure base cocaine (injected as the HCl salt) and was immediately placed in one compartment of the CPP box; or (2) received only a saline injection and was put in the other compartment. To emphasize, pretest-, training- and CPP test sessions were of equal duration, i.e., 15 min. Pretest bias for any of the two conditioning chambers was declared if during pretest the animal spent more time in one of the conditioning chambers. The initially non-preferred chamber was subsequently paired with the stimulus of interest (noncounterbalanced compartment allocation, see Zernig et al., 2013; Zernig and Pinheiro, 2015 for a detailed discussion). The allocation of the stimuli of interest to one of the two conditioning chambers (e.g., DSI to the horizontally wallpapered compartment and saline to the vertically wallpapered one) did not change during conditioning.

On experimental day 10, the CPP test was performed 24 h after the last conditioning trial by placing the mouse in the middle (neutral) compartment of the CPP apparatus and allowing it to freely move between the three compartments for 15 min. The preference for the stimulus of interest (DSI or coc) was then calculated as the time spent in the stimulus-associated compartment minus the time in the saline-associated compartment (given in seconds). The animal had to move in and out of the conditioning compartments at least five times during the CPP test for the data to be used for further analysis (which always was the case).

Reacquisiton/reexpression of DSI- or cocaine conditioning was tested by exposing the mouse to one 15 min episode of DSI or coc only (i.e., only one conditioning session) and then testing the mouse for CPP 24 h later.

The animals were assigned to one of five experimental groups. Their respective conditioning and testing protocols are shown in Figure 1.

#### Hierarchy Analysis: Scoring of Dominance vs. Subordination

The last of the four DSI episodes during CPP training was video-recorded and evaluated offline for signs of dominance/subordination in each mouse pair according to the scoring system by Bakker and colleagues (Veyrac et al., 2011) which is based on the level of aggression of the mice toward each other: Aggressive dominance (a hierarchy score of h3) was defined as three consecutive attacks by one mouse (aggressive grooming, biting and chasing); passive dominance (a score of h2) was defined as consistent threatening displacement by one mouse including upright or sideways postures; subordinate behavior (score of h0) was defined as retreat or fleeing by one mouse including “on back” position and crouching, and a draw (a score of h1) was defined as no attacks or consistent displacement occurring on the part of either mouse. Although the scoring experimenters were instructed to ignore all previously collected information on the individual mice, the offline hierarchy analysis was performed by the same experimenter who had previously quantified the time spent by the respective mice in the subsequent CPP test, so blinding to the behavior in the subsequent CPP was not absolute. However, due to the large number of video recordings analyzed by each experimenter, actual blinding seems plausible in most of the cases.

### Data Analysis

**Figure 2:**
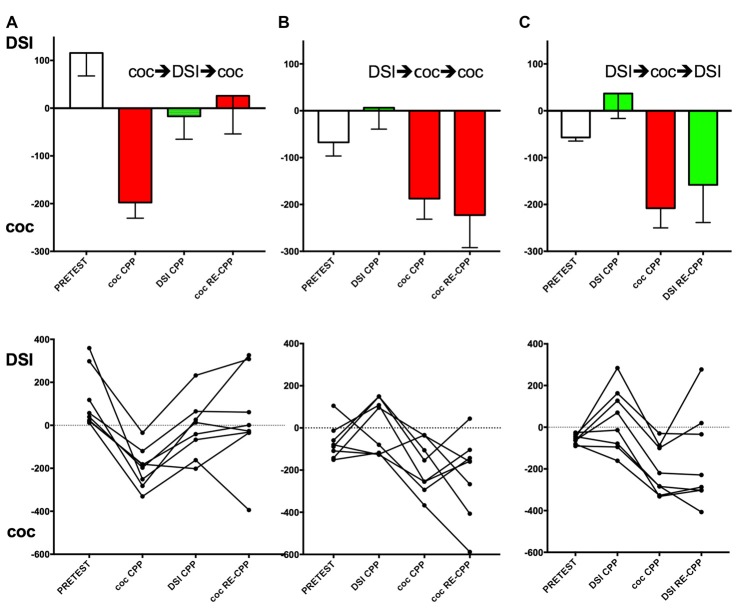
Effect of the conditioning history on subsequent CPP for DSI vs. cocaine. Group means and standard errors of the mean are shown in the top row, the trajectories of the individual mice are shown in the bottom row. Column **(A)**, group 1 (see Figure 1 for an outline of the experimental design); column **(B)**, group 2; column **(C)**, column, group 3. *X*-axis: coc CPP, cocaine CPP (red bars); DSI CPP, CPP for DSI (green bars); RE-CPP; reexpression of CPP 24 h after a final single conditioning session. Times (i.e., seconds) spent longer in the stimulus-associated compartment are shown on the *y*-axis, with positive values denoting a preference for DSI vs. saline and negative values indicating a preference for cocaine vs. saline. The first stimulus (DSI or coc) was always conditioned to the non-preferred compartment at pretest (open bars).

**Figure 3:**
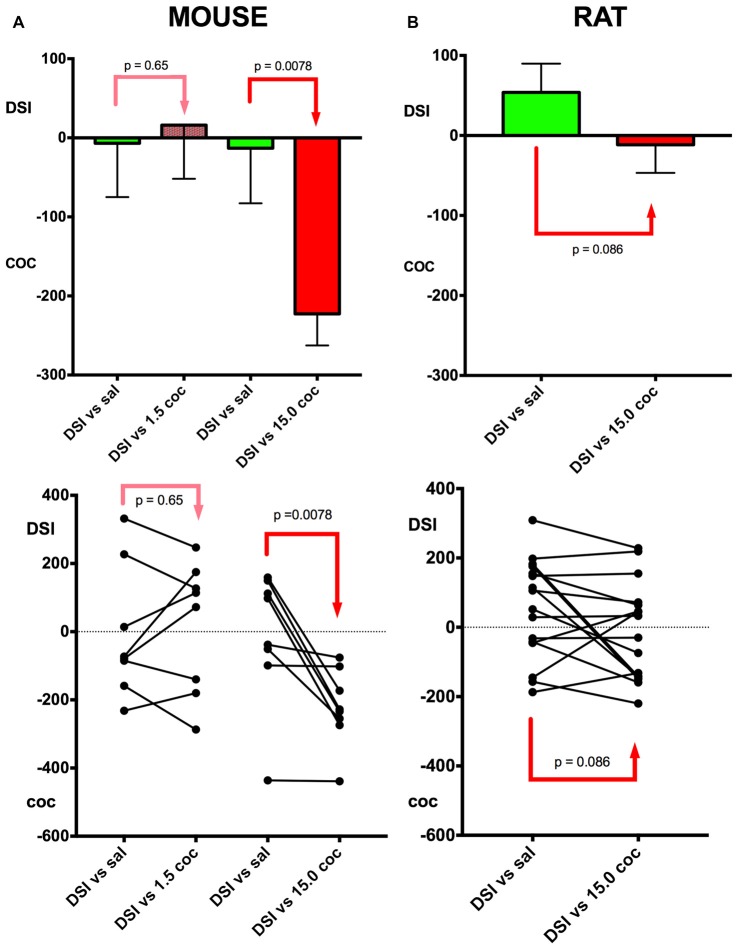
Relative preference for DSI vs. cocaine: Dose dependence and mouse vs. rat comparison. A schematic comparison of the conditioning of the different experimental groups is given in Figure 1. Column **(A)**, mouse data; column **(B)**, rat data. In group 4 (see Figure 1 for an outline of the experimental design), DSI was directly compared in previously DSI conditioned mice (DSI vs. sal) against 1.5 mg/kg i.p. cocaine (DSI vs. 1.5 coc); in group 5, against 15 mg/kg i.p. (DSI vs. 15.0 coc); both experiments were performed in C57BL/6J mouse. Group means and standard errors of the mean of the mouse experiments are shown in the top row, the trajectories of the individual animals are shown in the bottom row. *X*-axis: treatments. *Y*-axis: Times (i.e., seconds) spent longer in the respective stimulus-associated compartment, with positive values denoting a preference for DSI vs. saline or DSI vs. coc, and negative values indicating a preference for cocaine vs. saline or cocaine vs. DSI. DSI was always conditioned to the non-preferred compartment at pretest (not shown).

Data were first analyzed for normality with the D’Agostino and Pearson omnibus normality test. Of all the experimental groups and treatments tested, only the DSI/sal conditioning of group 2 (Figure 2B) and the DSI/sal conditioning of group 4 (Figure 3A) did not show normal distribution. However, for field conformity reasons, group averages are given as mean ± standard error of the mean (SEM). If all treatment effects of a group were normally distributed, statistical significance was tested with a one-way ANOVA followed by Tukey’s multiple comparisons test or, when testing a predefined hypothesis, a paired *t* test (parametric) or a Wilcoxon matched pairs signed rank test (nonparametric). If only one treatment did not show normal distribution, statistical significance was tested with the Friedman test followed by Dunn’s multiple comparisons test. Comparisons of individual groups were only performed if ANOVA or the Friedman test yielded overall significance (i.e., *p* < 0.05). All *p* values obtained in individual group comparisons refer to the two-sided version. In some cases, the behavioral changes are also shown at the level of the individual animal (trajectories). All statistical analyses were performed using Prism 7 for Mac OS X^1^.

## Results

Four conditioning sessions with 15 mg/kg i.p. cocaine produced a robust CPP for cocaine that was abolished by four conditioning sessions with DSI (Figure 2A). If these mice (group 1; see Figure 1 for a schematic timeline) were exposed to cocaine in its associated compartment for one time only, the reacquisition/reexpression of cocaine CPP was prevented at the group mean level (Figure 2A, top row; *p* = 0.013 for coc CPP vs. coc RE-CPP), confirming our initial findings obtained in rats (Fritz et al., 2011b). However, if DSI conditioning was followed by four conditioning sessions with cocaine (group 2, Figure 2B, top row), the mice showed full CPP for cocaine that remained the same if tested under the reacquisition/reexpression conditions (Figure 2B). If the mice were first conditioned to DSI (four exposures), full counterconditioning to cocaine was still obtained (Figure 2B, *p* = 0.016 for DSI CPP vs. coc CPP; Figure 2C, *p* < 0.0001 for DSI CPP vs. coc CPP). If cocaine-counterconditioned mice were finally tested for DSI reacquisition/reexpression after one exposure to DSI (group 3, Figure 2C), CPP for cocaine remained (Figure 2C, *p* = 0.36 for coc CPP vs. DSI RE-CPP).

Figure 2 (bottom row) also shows the individual animals’ trajectories. Corroborating previous results (Pinheiro et al., 2016) in which only 62% (i.e., 45 of 72) of C57BL/6J mice, i.e., “black six” mice from the Jackson (as opposed to the NIH) substrain, had shown individual preference for DSI (preceded by an i.p. saline injection) vs. an i.p. saline injection alone, previously naïve mice preferred DSI to a saline injection alone by 47% of the animals (i.e., 8 of 16 as shown Figures 2B,C and 7 of 16 as shown in Figure 3A, totalling 15 of 32) but avoided by the other 50%, resulting in a group mean around zero preference for DSI. In previously cocaine-conditioned animals, DSI counterconditioning resulted in the same 50% individual DSI preference (i.e., 4 of 8) vs. 50% individual DSI avoidance (Figure 2A). In contrast to the mixed preference for/avoidance of DSI-associated contextual stimuli, 100% (i.e., 24 of 24) of the C57BL/6J mice showed a CPP for cocaine (vs saline), regardless if they were naïve (Figure 2A) or had been conditioned to DSI first (Figures 2B,C).

Of 20 mouse pairs, only 1 pair had developed a hierarchy during the DSI conditioning, with one mouse having become subdominant according to quasi-blind observation of the fourth and last DSI encounter (i.e., showing consistent threating displacement of the other mouse including upright or sideways postures, given a hierarchy score h2 according to Veyrac et al., 2011) whereas the other mouse had developed subordinate behavior corresponding to an h0 score according to Veyrac et al. (2011) which is defined as “retreat or fleeing including “on back” position and crouching”. These findings corroborate previous results (Pinheiro et al., 2016). Because of the high percentage of hierarchically equal animals, we could not investigate how the hierarchy score was correlated with the subsequent CPP or CPA for DSI.

The dose dependence of CPP to cocaine was also tested in a concurrent CPP procedure, i.e., when pitching the cocaine stimulus directly against the DSI stimulus (groups 4 and 5, see Figure 1 for a schematic timeline). All C57BL/6J mice were first conditioned to DSI vs. saline and subsequently conditioned to DSI vs. cocaine in a concurrent CPP procedure (Figure 1; see also Zernig and Pinheiro, 2015) for a comparison of our different CPP-based experimental models). Previously DSI-conditioned mice did not change their preference when DSI was subsequently pitched directly against 1.5 mg/kg i.p. cocaine (*p* = 0.65, Figure 3A), whereas showed a 100% preference for cocaine if this 10-fold higher cocaine dose was directly pitched against DSI (*p* = 0.0078, Figure 3). In contrast to the C57BL/6J mice, only 50% (i.e., 8 of 16) Sprague-Dawley rats showed preference for 15 mg/kg cocaine when directly pitched against the DSI stimulus (Figure 3), regardless of the experimenter (TB vs. BP; not shown), confirming previous results obtained by different generations of experimenters (Fritz et al., 2011b; Kummer et al., 2014).

With respect to our continuing documentation and investigation of the experimenter effect in our paradigm (Kummer et al., 2014; Pinheiro et al., 2016), it should be noted that in the present study, no experimenter effect was seen between experimenter TB and BP when testing rats (bp = 0.67, not shown), also allowing to pool their data (Figure 3).

## Discussion

The present findings show that conditioning to DSI has to be long enough (i.e., four 15 min episodes in a cocaine-free environment) to countercondition previously cocaine-conditioned C57BL/6J mice and to protect them against the subsequent reacquisition/reexpression of cocaine CPP. We also demonstrated that even in DSI counterconditioned mice, a subsequent cocaine history that is long enough (i.e., four 15 min cocaine conditioning sessions) is sufficient to overcome the protective effect of DSI. Our findings also suggest that both the reversal of CPP from cocaine to DSI as well as that from DSI to cocaine, i.e., counterconditioning in either case, requires a learning process spanning four conditioning sessions (and not only a single one), regardless of the nature (i.e., cocaine as a prototypical drug of abuse vs. DSI as a physiologic stimulus) or the attractiveness of the initial stimulus (for critical evaluations of stimulus strength in various CPP procedures see e.g., Bardo et al., 1995, 2013; Bardo and Bevins, 2000; Tzschentke, 2007; Itzhak et al., 2014).

Our findings in mice corroborate with our initial findings in rats (Fritz et al., 2011b) in that four 15 min episodes of DSI abolished the previous cocaine CPP and prevented the reacquisition/reexpression of cocaine CPP (Figure 2A).

Our findings also corroborate previous findings (Kummer et al., 2014; Zernig and Pinheiro, 2015; Pinheiro et al., 2016) that DSI proved to be a stimulus that engenders very mixed responses in mice (as opposed to rats), i.e., preference for or avoidance of DSI-associated contextual stimuli in 48% (i.e., 19 of 40; Figures 2, 3) of the tested mice each, resulting in a group mean around zero preference for DSI. In contrast to DSI, 15 mg/kg i.p. cocaine engendered preference for cocaine-associated contextual stimuli in 100% (i.e., 32 of 32) of the C57BL/6J mice. Thus, 15 mg/kg i.p. cocaine proved to be a much more robustly rewarding stimulus than DSI in C57BL/6J mice. We also demonstrated dose dependence of cocaine CPP in a concurrent CPP procedure, i.e., when pitching the DSI stimulus directly against cocaine, as 1.5 mg/kg i.p. cocaine (as opposed to DSI) produced CPP in only 62% (i.e., 5 of 8) previously DSI-conditioned mice (Figure 3A), whereas 100% (i.e., 8 of 9; Figure 3A) of previously DSI-conditioned mice preferred 15 mg/kg, i.e., a 10-fold higher cocaine dose, to DSI, regardless of the experimenter handling and testing the rats, and confirming previous results obtained by different generations of experimenters (Fritz et al., 2011b; Kummer et al., 2014).

The above findings lead to the following consequences regarding the experimental design of future investigations on the neural basis of the orientation toward DSI vs. cocaine as a prototypical drug of abuse: (1) care must be taken to investigate the neural changes at the level of individual animal, dichotomizing results according to the individual mouse’s preference for vs. avoidance of the DSI-associated contextual stimuli. Analyzing any effect of DSI at the group mean level would give misleading, i.e., false negative, results. (2) In contrast to DSI, 15 mg/kg i.p. cocaine has consistently proved to be a supremely robust rewarding stimulus in C57BL/6J mice, engendering CPP in 100% of the animals, regardless if cocaine is compared to saline in the classic CPP procedure or pitched against DSI in the concurrent CPP procedure. Cocaine CPP also shows a clear dose dependence when pitched against DSI in a concurrent CPP procedure (present study and Kummer et al., 2014), increasing the amount of control the experimenter has with respect to the stimulus intensity—behavioral readout relationship. (3) Mice, by showing a mixed response to the DSI stimulus resulting in a group mean of roughly zero preference, are very different from Sprague-Dawley rats which show a much more pronounced preference for DSI, as consistently shown by both our group over several experimenter generations (Fritz et al., 2011a,b,c; Kummer et al., 2011, 2014, 2015; El Rawas et al., 2012a,b; Prast et al., 2012, 2014) and other independent groups using our paradigm (Yates et al., 2013) or other experimental approaches involving DSI (Peartree et al., 2012). These findings corroborate ample evidence that rats are more prosocial than mice (see e.g., refs in Kummer et al., 2014; Zernig and Pinheiro, 2015; Pinheiro et al., 2016) and the excellent work on social play in rats by Vanderschuren and colleagues (e.g., Trezza et al., 2010; Vanderschuren et al., 2016).

The fact that the CPP for a stimulus as attractive as 15 mg/kg cocaine could be counterconditioned with the—for C57BL/6J mice—much less uniformly attractive stimulus DSI (Figure 2A) was surprising; we had expected the cocaine conditioning to persist in the face of DSI conditioning in this rodent genus. Similarly, these initially cocaine-conditioned mice, after having been counterconditioned to DSI, did not reacquire/reexpress the initial CPP for cocaine after only one exposure to cocaine (Figure 2A).

We also want to emphasize again (see also Zernig and Pinheiro, 2015; Pinheiro et al., 2016) that the percentage of mice showing individual preference for DSI has decreased over several experimenter generations: while in our first study of DSI CPP in mice (summarized in Kummer et al., 2014), as many as 71% (i.e., 30 of 42) of C57BL/6N mice developed individual preference for DSI (and it has to considered that the NIH substrain was subsequently shown by us to be less prosocial than the currently used Jackson substrain), whereas in a later study (Pinheiro et al., 2016), only 62% (i.e., 45 of 72) of C57BL/6J mice showed individual preference for DSI. Finally, in the latest, i.e., present, study, the percentage of DSI preferring C57BL/6J decreased even further to 48% (i.e., 19 of 40). We have currently no explanation for this experimenter effect to offer (the breeder has always been the same, and a change in animal facilities did not impact on the systematic decrease in mice showing CPP for DSI) except that, plausibly, handling of the mice may have become more stressful over several experimenter generations. More stressful handling may plausibly bias the behavior of the mice against the whole CPP procedure, with a bigger impact on a less rewarding behavior like DSI and a much smaller impact on a strong reward like cocaine, as has been observed by us in a runway procedure (Crespo et al., 2006, 2008).

With respect to the likely success of investigating the effects of hierarchy/dominance/subordination (Zernig and Hiemke, 2017) in the C57BL/6J mouse substrain, consistent unilateral aggression or a degree of aggression corresponding to three or more attacks in a 15-min observation period was rarely observed in a previous (Pinheiro et al., 2016) as well as the present study, i.e., in only 3 of 52 pairs when pooled. Therefore, C57BL/6J mice alone offer little promise as an experimental species/genus to investigate phenomena like power abuse disorder (Zernig and Hiemke, 2017).

It may be argued that what we think is DSI counterconditioning simply represents extinction of coc CPP (for a detailed description of the psychologic constructs involved, see e.g., Zernig et al., 2007) because, at the group mean level, the DSI stimulus was no reward. However, in the present study, we did not add an extinction group as control. We had investigated the effect of extinction on subsequent reacquisition/reexpression of cocaine CPP in Sprague-Dawley rats (Fritz et al., 2011b) and, as all other aspects of the behavioral paradigms transferred so well from rat to mouse (see e.g., Kummer et al., 2015; Pinheiro et al., 2016), we thought that such an extinction control group was not ethically justified. Furthermore, at the level of the individual animal (Figure 2, bottom row), some mice in the present study showed a clear CPP for DSI, whereas some developed a clear aversion to DSI, either after a history of coc DSI (Figure 2, bottom row, left column) or before a history of coc DSI (Figure 2, bottom row, middle and right columns). To emphasize, regardless of previous aversion or preference for DSI, all 24 tested mice showed a conditioned preference for cocaine upon the completion of all four training sessions (Figure 2), whereas there was an equal (i.e., 12 vs. 12 mice) split for preference vs. aversion for DSI, again regardless of the previous conditioning history.

In conclusion, the present findings show that conditioning to DSI has to be long enough (i.e., four 15 min episodes in a cocaine-free environment) to countercondition previously cocaine-conditioned C57BL/6J mice and to protect them against the subsequent reacquisition/reexpression of cocaine CPP. However, a subsequent cocaine history that is long enough (i.e., four 15 min cocaine conditioning sessions) is sufficient to overcome the protective effect of DSI. Careful translation of our animal data on the human situation may indicate that currently abstinent but previously cocaine dependent individuals have to be exposed to drug-free social interaction, i.e., a non-drug alternative reinforcer, continuously for abstinence to be maintained. With respect to the experimental design of future investigations on the neural basis of the orientation toward DSI vs. cocaine as a prototypical drug of abuse, our findings allow the following conclusions: (1) care must be taken to investigate the neural changes at the level of individual animal, dichotomizing results according to the individual mouse’s preference for vs. avoidance of the DSI-associated contextual stimuli. Analyzing any effect of DSI at the group mean level would give misleading, i.e., false negative, results. (2) In contrast to DSI, 15 mg/kg i.p. cocaine has consistently proved to be a supremely robust rewarding stimulus in C57BL/6J mice. Cocaine CPP also shows a clear dose dependence when pitched against DSI in a concurrent CPP procedure, increasing the amount of control the experimenter has with respect to the stimulus intensity—behavioral readout relationship. (3) Mice, by showing a mixed response to the DSI stimulus resulting in a group mean of roughly zero preference, are very different from Sprague-Dawley rats which show a much more pronounced preference for DSI. Considering the notorious preference of human substance dependent individuals for the drug of abuse over drug-free social interaction (see e.g., Zernig et al., 2000, 2013; Nutt et al., 2010; Zernig and Pinheiro, 2015), the mouse may be translationally closer to humans than the rat.

## Author Contributions

TB, BSP and GZ designed the experiments with the support of RER. TB and BSP performed the experiments, GZ and TB analyzed the data. GZ and TB wrote the article. All coauthors provided instrumental manuscript input.

## Conflict of Interest Statement

The authors declare that the research was conducted in the absence of any commercial or financial relationships that could be construed as a potential conflict of interest.

## Footnotes

^1^www.graphpad.com

## References

[B1] Bardo M. T. Bevins R. A. (2000). Conditioned place preference: what does it add to our preclinical understanding of drug reward? Psychopharmacology 153, 31–43. 10.1007/s002130000569 11255927

[B2] Bardo M. T. Neisewander J. L. Kelly T. H. (2013). Individual differences and social influences on the neurobehavioral pharmacology of abused drugs. Pharmacol. Rev. 65, 255–290. 10.1124/pr.111.005124 23343975 PMC3565917

[B3] Bardo M. T. Rowlett J. K. Harris M. J. (1995). Conditioned place preference using opiate and stimulant drugs: a meta-analysis. Neurosci. Biobehav. Rev. 19, 39–51. 10.1016/0149-7634(94)00021-r 7770196

[B4] Crespo J. A. Stöeckl P. Zorn K. Saria A. Zernig G. (2008). Nucleus accumbens core acetylcholine is preferentially activated during drug- vs food reinforcement acquisition. Neuropsychopharmacology 33, 3213–3220. 10.1038/npp.2008.48 18418362

[B5] Crespo J. A. Sturm K. Saria A. Zernig G. (2006). Activation of muscarinic and nicotinic acetylcholine receptors in the nucleus accumbens core is necessary for the acquistion of drug reinforcement. J. Neurosci. 26, 6004–6010. 10.1523/JNEUROSCI.4494-05.2006 16738243 PMC6675236

[B6] El Rawas R. Klement S. Kummer K. K. Fritz M. Dechant G. Saria A.. (2012a). Brain regions associated with the acquisition of conditioned place preference for cocaine versus social interaction. Front. Behav. Neurosci. 6:63. 10.3389/fnbeh.2012.00063 23015784 PMC3449336

[B7] El Rawas R. Klement S. Salti A. Fritz M. Dechant G. Saria A.. (2012b). Preventive role of social interaction for cocaine conditioned place preference: correlation with FosB/DeltaFosB and pCREB expression in rat mesocorticolimbic areas. Front. Behav. Neurosci. 6:8. 10.3389/fnbeh.2012.00008 22403532 PMC3291868

[B8] Fritz M. El Rawas R. Klement S. Kummer K. Mayr M. J. Eggart V.. (2011a). Differential effects of accumbens core vs shell lesion in a rat concurrent conditioned place preference paradigm for cocaine vs social interaction. PLoS One 6:e26761. 10.1371/journal.pone.0026761 22046347 PMC3202564

[B9] Fritz M. El Rawas R. Salti A. Klement S. Bardo M. T. Kemmler G.. (2011b). Reversal of cocaine-conditioned place preference and mesocorticolimbic Zif268 expression by social interaction in rats. Addict. Biol. 16, 273–284. 10.1111/j.1369-1600.2010.00285.x 21309948

[B10] Fritz M. Klement S. El Rawas R. Saria A. Zernig G. (2011c). Sigma1 receptor antagonist BD1047 enhances reversal of conditioned place preference from cocaine to social interaction. Pharmacology 87, 45–48. 10.1159/000322534 21196793

[B11] Itzhak Y. Perez-Lanza D. Liddie S. (2014). The strength of aversive and appetitive associations and maladaptive behaviors. IUBMB Life 66, 559–571. 10.1002/iub.1310 25196552 PMC4175009

[B13] Kummer K. K. El Rawas R. Kress M. Saria A. Zernig G. (2015). Social interaction and cocaine conditioning in mice increase spontaneous spike frequency in the nucleus accumbens or septal nuclei as revealed by multielectrode array recordings. Pharmacology 95, 42–49. 10.1159/000370314 25592253

[B14] Kummer K. K. Hofhansel L. Barwitz C. M. Schardl A. Prast J. M. Salti A.. (2014). Differences in social interaction- vs cocaine reward in mouse vs rat. Front. Behav. Neurosci. 8:363. 10.3389/fnbeh.2014.00363 25368560 PMC4201146

[B12] Kummer K. Klement S. Eggart V. Mayr M. J. Saria A. Zernig G. (2011). Conditioned place preference for social interaction in rats: contribution of sensory components. Front. Behav. Neurosci. 5:80. 10.3389/fnbeh.2011.00080 22232578 PMC3246900

[B15] Nutt D. J. King L. A. Phillips L. D. (2010). Drug harms in the UK: a multicriteria decision analysis. Lancet 376, 1558–1565. 10.1016/s0140-6736(10)61462-6 21036393

[B16] Peartree N. A. Hood L. E. Thiel K. J. Sanabria F. Pentkowski N. S. Chandler K. N.. (2012). Limited physical contact through a mesh barrier is sufficient for social reward-conditioned place preference in adolescent male rats. Physiol. Behav. 105, 749–756. 10.1016/j.physbeh.2011.10.001 22008744 PMC3975131

[B17] Pinheiro B. S. Seidl S. S. Habazettl E. Gruber B. Bregolin T. Zernig G. (2016). Dyadic social interaction of C57BL/6 mice vs interaction with a toy mouse: conditioned place preference/aversion, substrain differences and no development of a hierarchy. Behav. Pharmacol. 27, 279–288. 10.1097/FBP.0000000000000223 26905190 PMC4780246

[B18] Prast J. M. Kummer K. K. Barwitz C. M. Humpel C. Dechant G. Zernig G. (2012). Acetylcholine, drug reward and substance use disorder treatment: intra- and inerindividual striatal and accumbal neuron ensemble heterogeneity may explain apparent discrepant findings. Pharmacology 90, 264–273. 10.1159/000342636 23018268

[B19] Prast J. M. Schardl A. Schwarzer C. Dechant G. Saria A. Zernig G. (2014). Reacquisition of cocaine conditioned place preference and its inhibition by previous social interaction preferentially affect D1-medium spiny neurons in the accumbens corridor. Front. Behav. Neurosci. 8:317. 10.3389/fnbeh.2014.00317 25309368 PMC4174134

[B20] Trezza V. Baarendse P. J. Vanderschuren L. J. (2010). The pleasures of play: pharmacological insights into social reward mechanisms. Trends Pharmacol. Sci. 31, 463–469. 10.1016/j.tips.2010.06.008 20684996 PMC2946511

[B21] Tzschentke T. M. (2007). Measuring reward with the conditioned place preference (CPP) paradigm: update of the last decade. Addict. Biol. 12, 227–462. 10.1111/j.1369-1600.2007.00070.x 17678505

[B22] Vanderschuren L. J. Achterberg E. J. Trezza V. (2016). The neurobiology of social play and its rewarding value in rats. Neurosci. Biobehav. Rev. 70, 86–105. 10.1016/j.neubiorev.2016.07.025 27587003 PMC5074863

[B23] Veyrac A. Wang G. Baum M. J. Bakker J. (2011). The main and accessory olfactory systems of female mice are activated differentially by dominant versus subordinate male urinary odors. Brain Res. 1402, 20–29. 10.1016/j.brainres.2011.05.035 21683943 PMC3155078

[B24] Whishaw I. Q. Metz G. A. S. Kolb B. Pellis S. M. (2001). Accelerated nervous system development contributes to behavioral efficiency in the laboratory mouse: a behavioral review and theoretical proposal. Dev. Psychobiol. 39, 151–170. 10.1002/dev.1041 11745309

[B25] Yates J. R. Beckmann J. S. Meyer A. C. Bardo M. T. (2013). Concurrent choice for social interaction and amphetamine using conditioned place preference in rats: effects of age and housing condition. Drug Alcohol Depend. 129, 240–246. 10.1016/j.drugalcdep.2013.02.024 23540449 PMC3628407

[B26] Zernig G. Ahmed S. H. Cardinal R. N. Morgan D. Acquas E. Foltin R. W.. (2007). Explaining the escalation of drug use in substance dependence: models and appropriate animal laboratory tests. Pharmacology 80, 65–119. 10.1159/000103923 17570954

[B29] Zernig G. Giacomuzzi S. Riemer Y. Wakonigg G. Sturm K. Saria A. (2003). Intravenous drug injection habits: drug users’ self-reports versus researchers’ perception. Pharmacology 976, 49–56. 10.1159/000068731 12660479

[B27] Zernig G. Hiemke C. (2017). Making the case for ‘power abuse disorder’ as a nosologic entity. Pharmacology 100, 50–63. 10.1159/000475600 28467994 PMC5872562

[B30] Zernig G. Kummer K. K. Prast J. M. (2013). Dyadic social interaction as an alternative reward to cocaine. Front. Psychiatry 4:100. 10.3389/fpsyt.2013.00100 24062696 PMC3770939

[B31] Zernig G. Saria A. Kurz M. O’Malley S. S. (2000). Handbook of Alcoholism. Boca Raton, FL: CRC Press.

[B28] Zernig G. Pinheiro B. S. (2015). Dyadic social interaction inhibits cocaine-conditioned place preference and the associated activation of the accumbens corridor. Behav. Pharmacol. 26, 580–594. 10.1097/FBP.0000000000000167 26221832 PMC4523229

